# Dissimilar flexibility of α and β subunits of human adult hemoglobin influences the protein dynamics and its alteration induced by allosteric effectors

**DOI:** 10.1371/journal.pone.0194994

**Published:** 2018-03-27

**Authors:** Gusztáv Schay, András D. Kaposi, László Smeller, Krisztián Szigeti, Judit Fidy, Levente Herenyi

**Affiliations:** Department of Biophysics and Radiation Biology, Semmelweis University, Budapest, Hungary; Russian Academy of Medical Sciences, RUSSIAN FEDERATION

## Abstract

The general question by what mechanism an “effector” molecule and the hemes of hemoglobin interact over widely separated intramolecular distances to change the oxygen affinity has been extensively investigated, and still has remained of central interest. In the present work we were interested in clarifying the general role of the protein matrix and its dynamics in the regulation of human adult hemoglobin (HbA). We used a spectroscopy approach that yields the compressibility (*κ*) of the protein matrix around the hemes of the subunits in HbA and studied how the binding of heterotropic allosteric effectors modify this parameter. *κ* is directly related to the variance of volume fluctuation, therefore it characterizes the molecular dynamics of the protein structure. For the experiments the heme groups either in the α or in the β subunits of HbA were replaced by fluorescent Zn-protoporphyrinIX, and series of fluorescence line narrowed spectra were measured at varied pressures. The evaluation of the spectra yielded the compressibility that showed significant dynamic asymmetry between the subunits: *κ* of the α subunit was 0.17±0.05/GPa, while for the β subunit it was much higher, 0.36±0.07/GPa. The heterotropic effectors, chloride ions, inositol hexaphosphate and bezafibrate did not cause significant changes in *κ* of the α subunits, while in the β subunits the effectors lead to a significant reduction down to 0.15±0.04/GPa. We relate our results to structural data, to results of recent functional studies and to those of molecular dynamics simulations, and find good agreements. The observed asymmetry in the flexibility suggests a distinct role of the subunits in the regulation of Hb that results in the observed changes of the oxygen binding capability.

## Introduction

**Fig 1:**
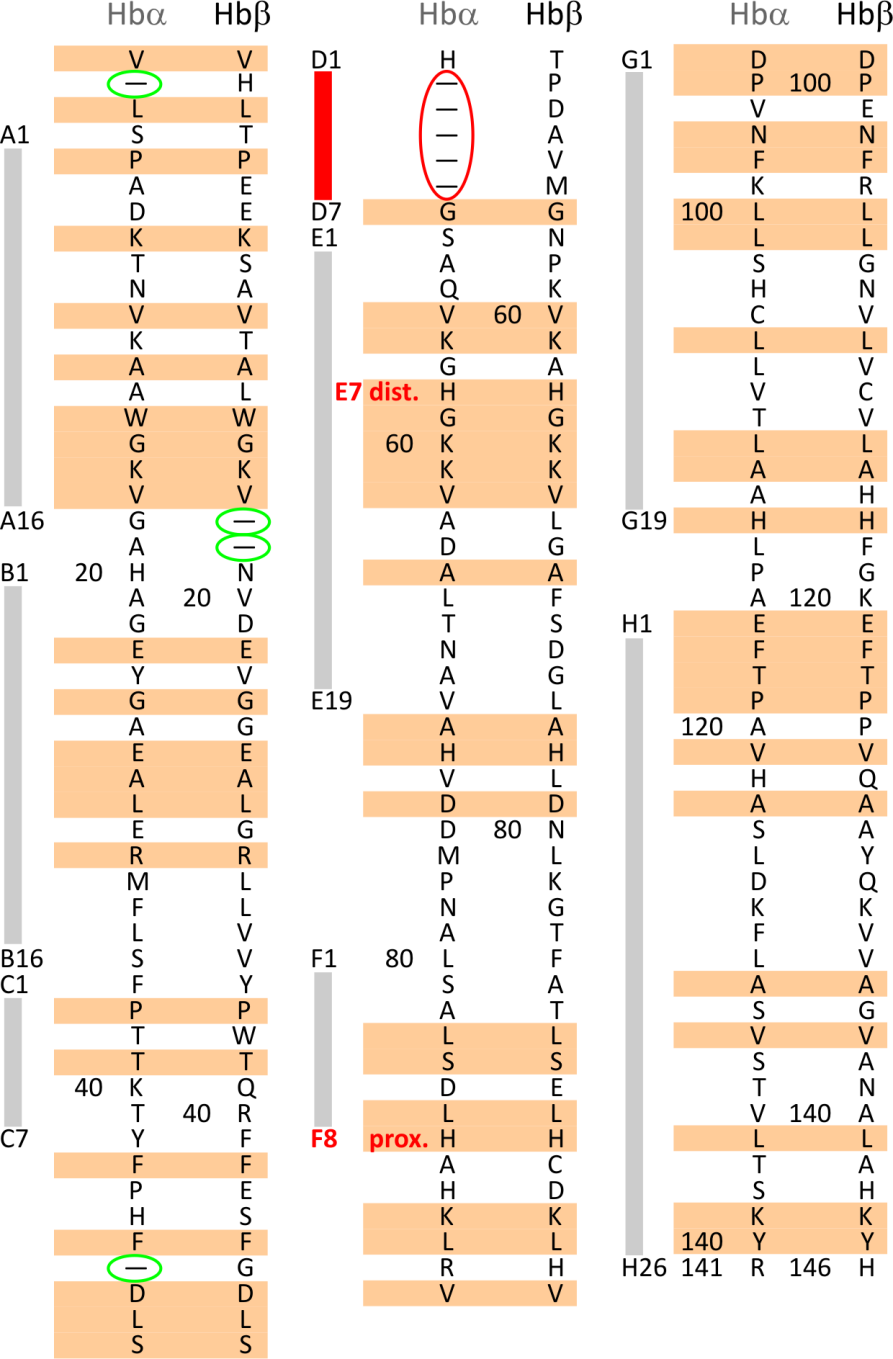
Amino acid sequence of hemoglobin (HbA) in α and β subunits. α-helices are denoted by capital letters (with numbers) from A to H. Minor deviations because of the absence of amino acid are marked with green ellipses, the major, dominant difference marked with red ellipse in D helix. Distal (E7) and proximal (F8) histidines (H) are also denoted.

**Fig 2:**
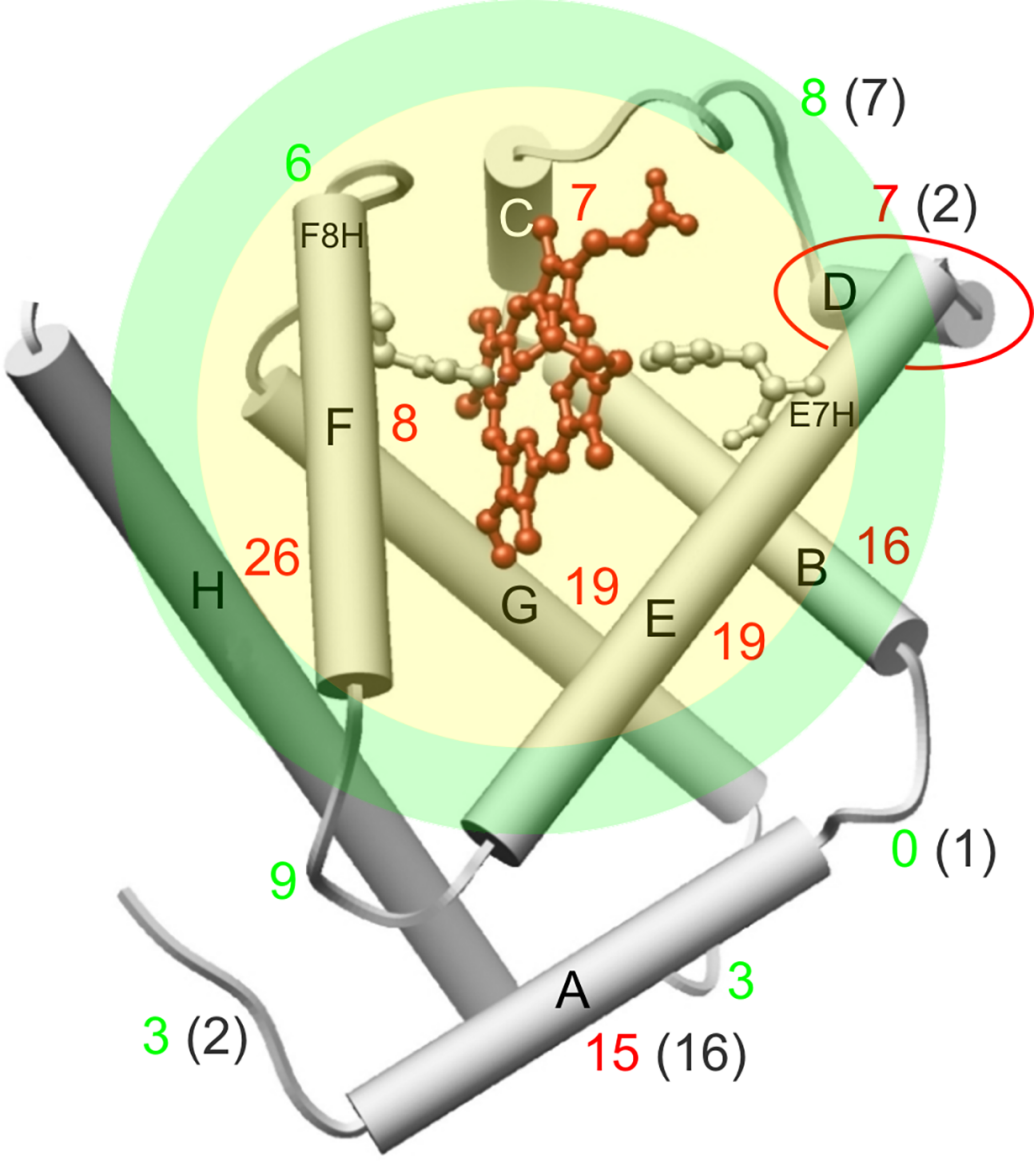
Structure of β subunits of hemoglobin (β-HbA). α-helices are shown as cylinders, number of amino acids of segments is marked with colored digits: red for α-helices, green for loops (gray in the brackets refers to α subunit). The heme group is stabilized by the distal (E7H) and proximal (F8H) histidines. Helix D is marked with red ellipse as the dominant difference between β and α subunits [6]. The transparent colored discs denote the estimated vicinity of the heme of which is characterized by the compressibility in the present work. Yellow and green area mean the spheres with radius *R*_98%_ and *R*_99%_, labelling a rage of estimation with 2% and with 1% error respectively (see Appendix II).

Allosteric effect is a widely used term in biochemistry and biophysics that means in general the functional modulation of an active site brought about by the binding of ligands at a distant site on the same molecule [1, 2]. These ligands are called “allosteric effectors”. If they are identical with the basic ligand of the protein we use the term “homotropic” effectors, in other cases they are called “heterotropic” effectors. Cooperativity of oxygen binding in this context is a homotropic allosteric effect. Hemoglobin that is essential in binding, transporting and offloading oxygen from the lungs to the tissues to respiring cells is one of the most widely studied biomolecules. Human adult hemoglobin (HbA) is the classic example of an allosteric protein. HbA is a homo-dimer (α_1_β_1_)(α_2_β_2_) of two hetero-dimers, (αβ). Each of the monomers consists of one polypeptide chain, α of 141, β of 146 amino acid residues forming 7 and 8 α-helices, respectively (named A to H, but helix D in β chains has no equivalent in the α subunits). Each subunit contains one protoheme IX as the prosthetic group, to which one O_2_ can bind reversibly [3–5] (see Figs 1 and 2). O_2_ binding to one prosthetic group is known to be allosterically regulated both by O_2_ binding to that of another subunit (homotropic effect), and by binding specific other molecules (heterotropic effect).

Several models have been developed to explain the function of Hb. Here we just give a short summary as an enumeration however the reader can find a longer but concise overview at the end of the paper (see Appendix III).

The first classic model has been the “two-state allosteric model” (or MWC model) of Monod, Wyman, and Changeau [7–9] which was structurally interpreted by Perutz in his “stereochemical model” [10]. The “sequential model” (or KNF model) of Koshland, Nemethy and Filmer [11] meant another approach to the interpretation of allostery. The newer “ensemble allostery model” (EAM) combined the earlier ones [12] and a recent article emphasizes the chemical perspective on allostery [13].

Based on the literature we conclude that the global mechanism is similar in the case of homotropic and heterotropic effects. In connection with heterotropic effects some inconsistencies occurred [14] but the “tertiary two state model” (TTS model) managed to solve the problem [15]. According to another approach some data initiated the reformulation of the earlier models by the “global allostery model” [16, 17]. Earlier in our research group it was also demonstrated both by an experimental technique and by computational modeling, that conformational changes induced by the binding of the heterotropic allosteric effectors Chloride ions (Cl^-^), Inositol Hexaphosphate (IHP) and Bezafibrate (BZF), influenced the interdimeric interfaces of Hb in both conformational states, supporting the global allostery model [18, 19]. The detailed mechanism of structural rearrangements was also studied by the Perahia group [20, 21].

Finally, we have to mention the work of Tsai and Nussinov, they presented a “unified view about allostery” [22, 23].

**Fig 3:**
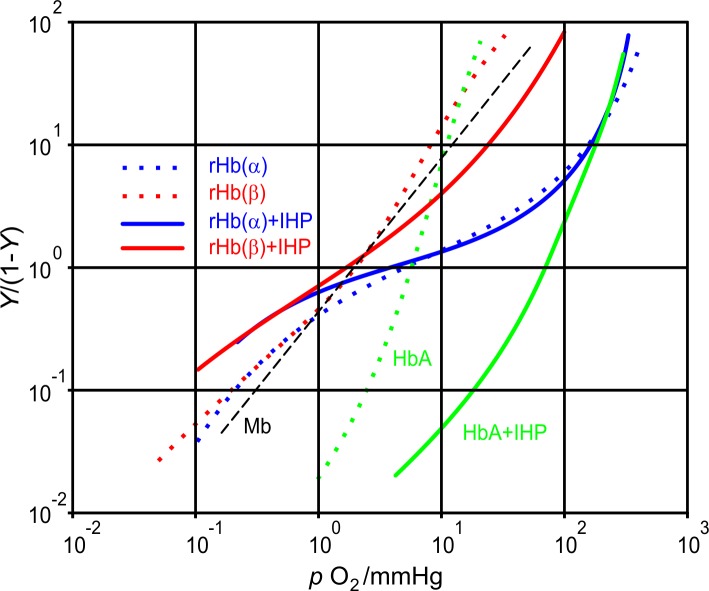
O_2_ binding curves (Hill plots) of cavity mutants and native human HbA in the absence and presence of IHP (dotted and continues lines respectively), and that of sperm whale Mb (dashed straight line): rHb(α) in blue, rHb(β) in red, HbA in green color. (Data adapted from Ref. [25].) *Y* is the fractional oxygen saturation and *p*O_2_ is the partial pressure of oxygen, pH = 7.4. Tangent of the curves is characterized by two parameters: the slope of the straight line being the Hill coefficient and the intercept with the *Y/*(1-*Y*) = 1 line (*p*_50_) being proportional to the apparent dissociation constant.

Besides the global symmetry of Hb a structural asymmetry of α and β subunits is conspicuous. Recently an interesting functional difference was demonstrated. Using resonance Raman spectroscopy in silica gel, which greatly slows protein motions, the evolution of the Fe-His stretching frequency after CO photodissociation (which is a monitor of heme reactivity from state R to T) is faster for β(Fe-His) than for α(Fe-His) [24]. Another research group showed distinct roles of α and β subunits in O_2_ binding with the help of cavity mutant HbA-s [25]. The Hill plots of the two mutants, in which the Fe-His bond was cleared is very different (Fig 3). The α mutant of recombinant Hb (rHb(α)) has biphasic O_2_ binding, while the β mutant (rHb(β)) is locked into more or less one state, similarly to Mb. The authors also pointed out, that the detachment of the F-helix from the heme in the β subunits causes an increase of O_2_ affinity in the α subunits whose hemes are attached to the F-helix.

If conformational dynamics play decisive role in Hb allostery, and experimental data suggest an asymmetric role of the α and β subunits, then this asymmetry should be observable more directly. To prove this hypothesis, we applied an experimental approach to this problem that resulted in the characterization of subunit dynamics as it became modified by binding heterotropic allosteric effectors. The selected technique has been pressure tuning Fluorescence Line Narrowing spectroscopy (FLN), and by using proper hybrid Hb samples, the dynamics of the subunits could be separately studied and characterized by the compressibility of the protein matrix around the heme group.

### Pressure tuning FLN spectroscopy for compressibility determination

Fluorescence line narrowing (FLN) spectroscopy is a high-resolution, laser excitation spectroscopy technique performed at cryogenic temperatures. (For general discussion of this technique see relevant papers and reviews [26–28]. A short summary is given in Appendix I.) It has been shown that the method may also be successfully used when the matrix surrounding the fluorophore is a protein [29, 30]. The outcome of an FLN experiment is a series of fluorescence spectra resolved for vibronic transitions, and the evaluation yields the so called inhomogeneous distribution function (IDF) of the (0, 0) electronic transition energy (see Appendix I). The spectral broadening of this distribution function is caused by the inhomogeneity of the matrix around the fluorophore, in our case the slightly different local structure of hybrid Hb molecules around the modified heme. This inhomogeneity arises from the changing interaction between the chromophore and its environments due to the continuous temporal random fluctuation of the protein. A sudden cooling of the protein solution to cryogenic temperatures will freeze the chromophore environments into their present status and this ensemble of protein molecules can be characterized by the IDF.

We applied this technique combined with pressure tuning to obtain information about the dynamics of hemoglobin subunits. The fluorophores were Zn-protoporphyrinIX molecules replacing the heme either in the α or in the β subunits. Oxygen equilibrium studies and x-ray crystallography have shown that this derivative, as a five-coordinated metal hybrid of HbA, can be considered analogous to T-state HbA [31]. According to the basics of statistical physics (see for example Ref. [32]). the variance of the volume fluctuations of a system–arising from conformational dynamics in a protein–is directly related to its compressibility *κ*:

$$\langle {\left(V-\langle V\rangle \right)}^{2}\rangle =\kappa \;kT\langle V\rangle ,$$ (1)

where *V* is the volume, *T* is the temperature, *k* is the Boltzmann-constant and angle brackets denote the expected value (averaging). It can be shown that FLN measurements performed at varied hydrostatic pressures may yield the *κ* of the matrix (protein) surrounding the fluorophore. Since the sample is at cryogenic temperatures, in the frozen state denaturation is not expected to occur, even at very high pressures, because the surface of the protein is firmly bound to a solid matrix. As a consequence of pressurization, a shift and a broadening can be observed on the IDF of the fluorophore determined by FLN, and *κ* can be calculated from these changes as follows. The pressure-induced shift of a specific electronic transition was theoretically interpreted by Laird and Skinner [33]. According to their theory, the frequency of a spectral line of an optical transition under pressure *p* is given by the equation:

$$\nu_{p}=\nu_{0}+2(\nu_{0}-\nu_{\mathrm{vac}})\kappa p=\nu_{0}+2\nu_{s}\kappa p$$ (2)

where *ν*_0_ is the frequency of a spectral line at normal pressure, *ν*_vac_ is the so called vacuum frequency, where the chromophore would absorb light in the absence of the matrix. *ν*_s_, the difference of *ν*_0_ and *ν*_vac_ is called as the solvent shift. This theory was generalized by Smeller and Fidy for IDF-s [34]. Assuming normal distribution, the shift and the corresponding broadening of the IDF can be calculated:

$$\nu_{0p}=\nu_{00}+2\kappa p(\nu_{0\;s})$$ (3)

$$\sigma_{p}=(1+2\kappa p)\sigma_{0}$$ (4)

where the extra “0”-s in the indices of *ν* emphasize the fact that the parameter corresponds to the center of the normal distribution, *𝜎* is the width of the normal distribution. It is noticeable that the center of the IDF shifts under pressure the same way as the individual spectral lines do. The slope of both the shift and the broadening on the frequency scale is proportional to the *κ*, and thus to the extent of volume fluctuation of the measured system (hemoglobin subunits).

Here we have to make two remarks. While in the outlined technique the compressibility is determined at cryogenic temperatures, we think that this parameter is a rather good approximation for the compressibility at room temperature. It is reasonable to suppose that the IDF accounts for almost all states of conformational fluctuations of room temperature, but in “frozen” phase. We measure the pressure effect on this conformational population, and think that it should not be different when the motions are activated and the elements of the distribution are switching between each other at room temperature. Experimental results obtained on the protein horseradish peroxidase showed excellent agreement between the compressibility obtained by FLN at 10 K and that obtained by ultrasound velocity technique at room temperature [34].

We also have to remark that in the case of Hb the compressibility as global parameter measured by the present technique is characteristic for the protein matrix near the heme group and not for longer distance. Nevertheless, there is a crucial question: can we determine more precisely what is the range of chromophores’ interaction with the environment? We may get a lower limit from Ref. [35] for cytochrome c, but the estimation based on hole-burning experiments, where the applied pressure is much smaller than in pressure tuning FLN. Thus we take a different approach in Appendix II to estimate the interaction volume “visible” to the reporter chromophore in FLN experiments. From this estimation we can assume that the compressibility values are referring to approximately the size of a single monomer (see the transparent colored discs in Fig 2).

In this paper we show that this method is capable of yielding an experimental parameter (*κ*) related to protein conformational dynamics and our presented data clearly support the suggested asymmetry in the role of the α and β subunits in Hb allostery and in the influence of heterotropic allosteric effectors. We think that these data unravel the structural background of other experimental observations and serve as new experimental support for the results of molecular dynamics simulations.

## Materials and methods

### Chemicals

Chemicals such as sodium chloride (NaCl), 4-(2-hydroxyethyl)-1-piperazineethanesulfonic acid (HEPES), inositol hexaphosphate (IHP), and bezafibrate (BZF), the highest purity available, were purchased from Sigma-Aldrich. All samples were prepared in 100 mM HEPES, pH 7.4 with double distilled water. IHP was used in a final concentration of 2 mM, BZF in 10 mM, and the NaCl concentration was 100 mM. All the allosteric effectors were added in a concentration to ensure saturation of the binding sites (of 60 μM protein).

### Sample preparation

The experiments were performed on hybrid human HbA with Zn-PP substituting the heme either in the α or in the β subunits (Zn-HbA) that were prepared in Prof. T. Yonetani’s laboratory at University of Pennsylvania (Philadelphia, USA) as described in Ref. [17], and kindly provided for our experiments. Samples were stored at -80°C and their quality after thawing was checked before further experiments by recording an absorption spectrum in the 270–700 nm wavelength range. Only samples with no detectable deterioration were used. All samples were adjusted to 60 μM final Zn-HbA concentration in 50% v/v glycerol-buffer solution (pH = 7.4) before measurement. The sample quality was controlled by absorption spectroscopy after each experiments at ambient pressures and temperatures and no significant changes were detected due to the addition of various effectors or the pressure changes.

### Experimental setup

For the FLN experiment a Coherent 899–01 ring dye laser pumped by a Coherent Innova 307 Ar-ion laser was used. The dye used for excitation was Rhodamine 560 (Exciton Laser Dyes). The spectral width of the laser line was 0.7 cm^-1^, the output power was 1 mW. Fluorescence emission was collected at approximately 35° angle relative to excitation and focused into a THR 1000 monochromator (Jobin-Yvon). For detection a CCD camera (WR CCD-4679 DV401-BV, Andor Technology) cooled to -65° C was used, mounted at the exit slit of the monochromator with a final resolution better than 1 cm^-1^ in the whole measured spectral range. Integration time was 40 s for each spectrum. The sample was maintained at 10 K inside a diamond anvil cell with a closed cycle M22 cryostat (Cryophysics SA) equipped with a Model-330 auto-tuning temperature controller (Lake Shore Inc.). Typical pressure inside the cryostat was 10^−5^ bar, it was monitored with a model AGD-101-L Pirani-gauge (Edwards High Vacuum International), and was generated with a rotary vacuum pump (model RV5, Edwards High Vacuum International). Spectra were analyzed by a custom-designed software package (“IdfFit”), which also allow for the wavenumber-calibration of the CCD camera. Pressure was adjusted with helium gas in the special low-temperature diamond anvil cell (Diacell Products). This cell of 40 nl volume allows for applying pressures in the range of 0–1.5 GPa. Small ruby crystals inside the frozen sample were used for the pressure determination [36]. The accuracy of the pressure values measured at low temperatures was typically 0.02 GPa.

### Evaluation of FLN spectra, determination of the IDF and compressibility

To obtain the compressibility, series of FLN spectra were measured, each at varied excitation frequencies and all series at varied pressures. The highest intensity part of spectra is composed of (0, 0) transition lines (see Appendix I). The first step of evaluation is the baseline-correction removing the non-resolved or non-resonant background from the spectra (see Appendix I). Based on the energy difference of excitation and (0, 0) emission energies (called as vibrational energy) one may select a line from each spectrum that belongs to the same vibrational state. After this, one needs to track such a selected spectral line at varied excitation wavenumbers and get a reading of its intensity as a function of its (0, 0) emission energy, giving a possible representation of the IDF. Selecting another line and plotting the results, the same kind of IDF will be represented with the only difference that due to the different line intensity the total area under the curve varies, being a consequence of the different transition probabilities of the excitation (see Appendix I). The inverse of the corresponding area was used as a weighting factor for normalization. The reading of line intensities was performed by using the “Peakfinder” component of the custom-designed software package (“IdfFit”). The spectral analysis was performed at each adjusted pressure value. The IDFs were fitted by Gaussians. The center and the width values were plotted as functions of pressure and the compressibility was determined according to Eqs 3 and 4.

## Results

### FLN spectra and IDF of hybrid HbAs

**Fig 4:**
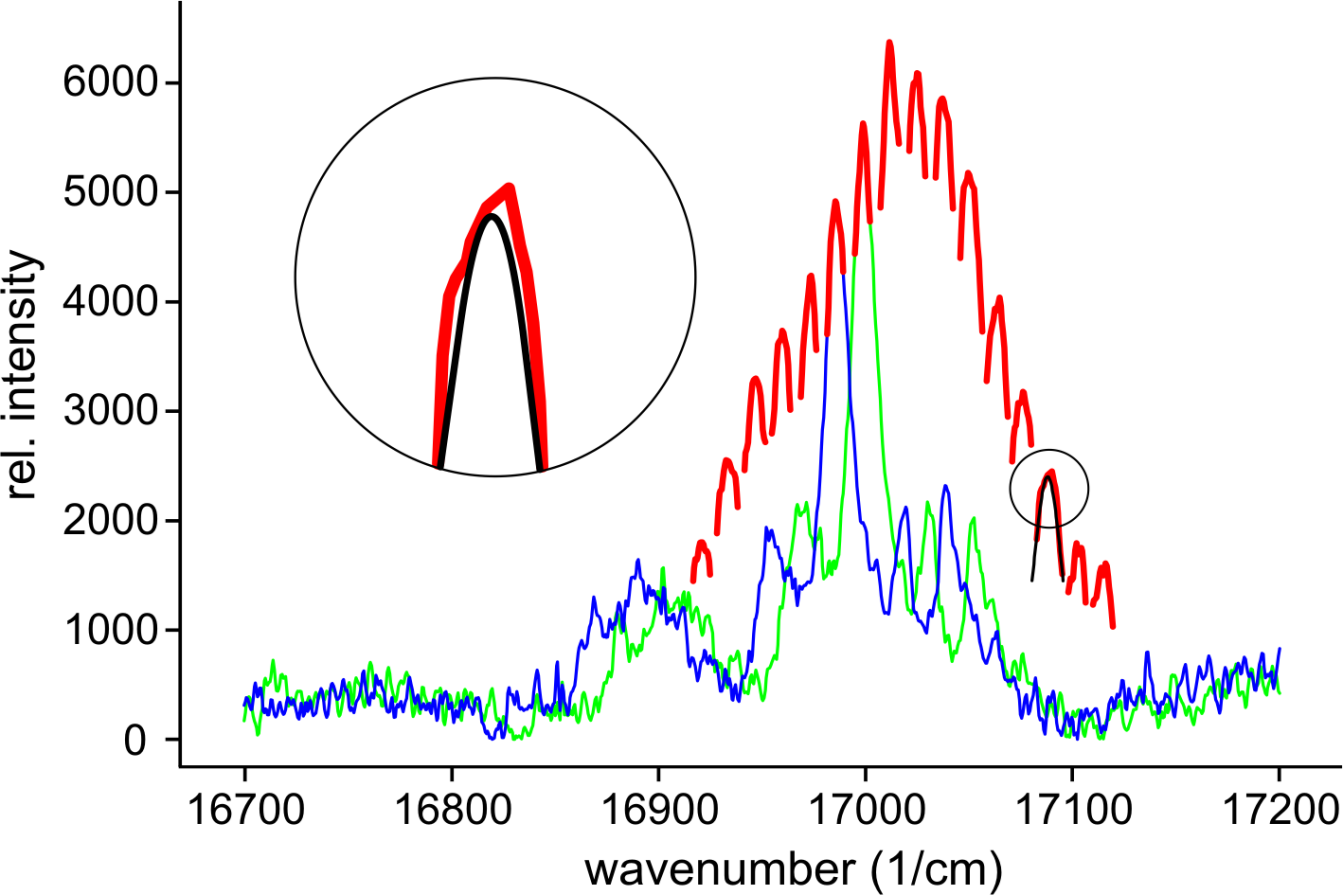
Fluorescence line narrowing (FLN) spectra of β-Zn-HbA. Blue and green curves show spectra at 0.14 GPa pressure and at two different excitation frequencies: 17958 cm^-1^ and 17971 cm^-1^ respectively; the detectable emission wavenumber range of the CCD camera is 16700–17200 cm^-1^. The changing peak maximum of the most intense spectral line (*ν*_vibratinal_ = 973 cm^-1^) marked with red color is also shown as it becomes shifted by the distinct excitation frequencies in the 17893–18089 cm^-1^ wavenumber range. Inset shows a fitted peak (black) determined by the “Peakfinder” software.

In Fig 4 two FLN spectra of β-Zn-HbA are presented marked with blue and green colors. The difference between the two curves is that the excitation frequencies were different by about 13 cm^-1^. If the excitation difference is small, comparable to the linewidth, the shape of the two curves is rather similar, just shifted as much as the excitation frequency changed. The relative intensities of the shifted spectral lines are, however, somewhat different. In one spectrum each line corresponds to a specific vibrational state with a characteristic vibrational frequency. Let us choose one of the spectral lines, for example the most intense one. In Fig 4 the vibrational frequency of this line for both spectra is 973 cm^-1^. The change of the intensity of such a line with the excitation frequency is a consequence of the fact that at different excitation frequencies, different number of molecules can be excited to the same level. Therefore, the intensity change follows the inhomogeneous distribution of the (0, 0) transition energies. The change in the peak maximum of the selected line in a broader excitation frequency range is marked with red color in Fig 4. The somewhat noisy measured peak functions were replaced by fitted peaks for the evaluation of line intensities as shown in the inset.

**Fig 5:**
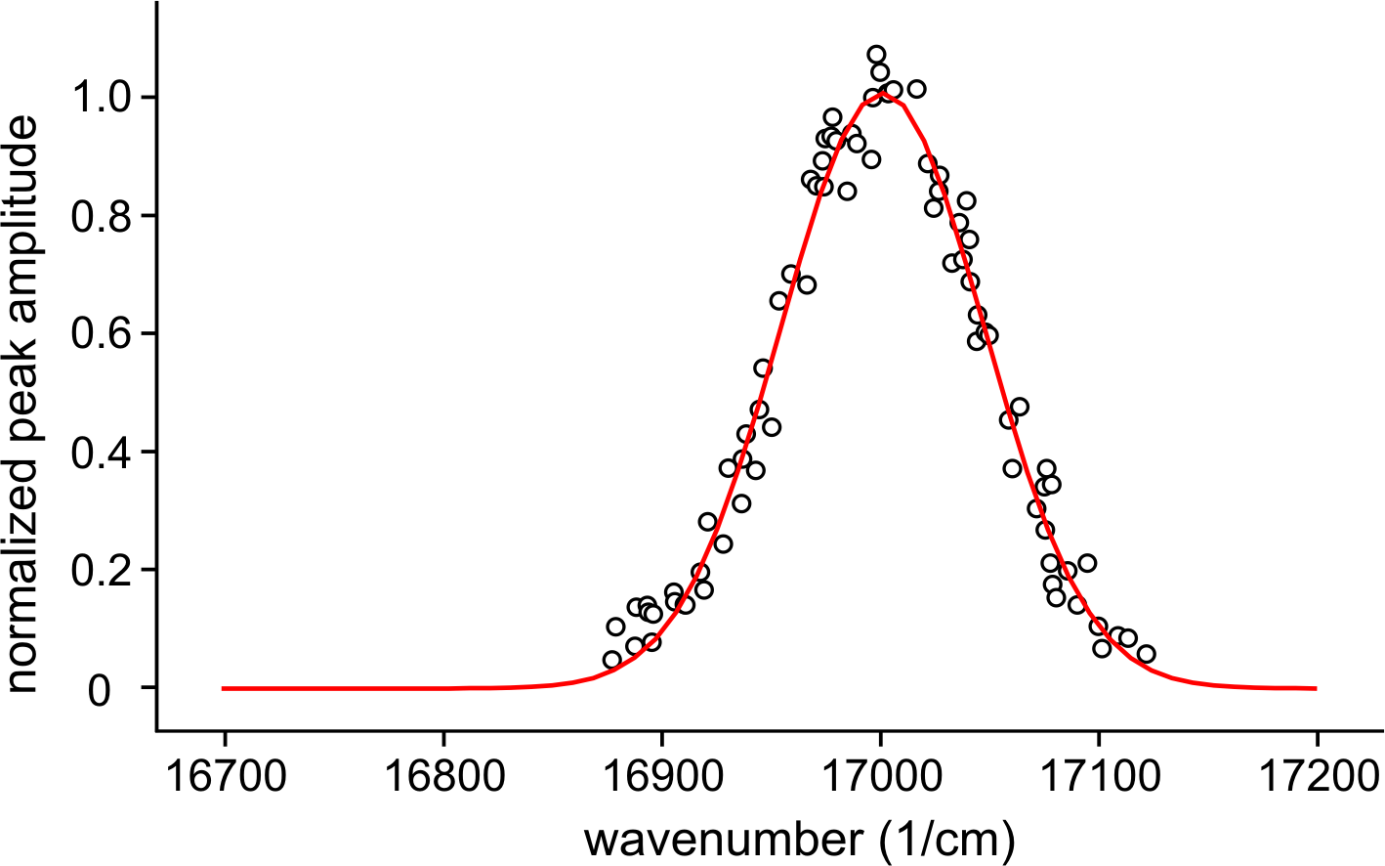
Determination of inhomogeneous distribution function (IDF). Normalized amplitudes of the peaks determined from fluorescence line narrowing (FLN) spectra of β-Zn-HbA after a background correction as a function of their center in the 16875–17125 cm^-1^ wavenumber range with a fitted Gaussian as an inhomogeneous distribution function (IDF).

Fig 5 shows the result of spectral evaluation in the case of β-Zn-HbA. The IDFs obtained by various selected vibronic excitation data were unified after normalization and fitted with Gaussian.

### IDF as function of pressure

**Fig 6:**
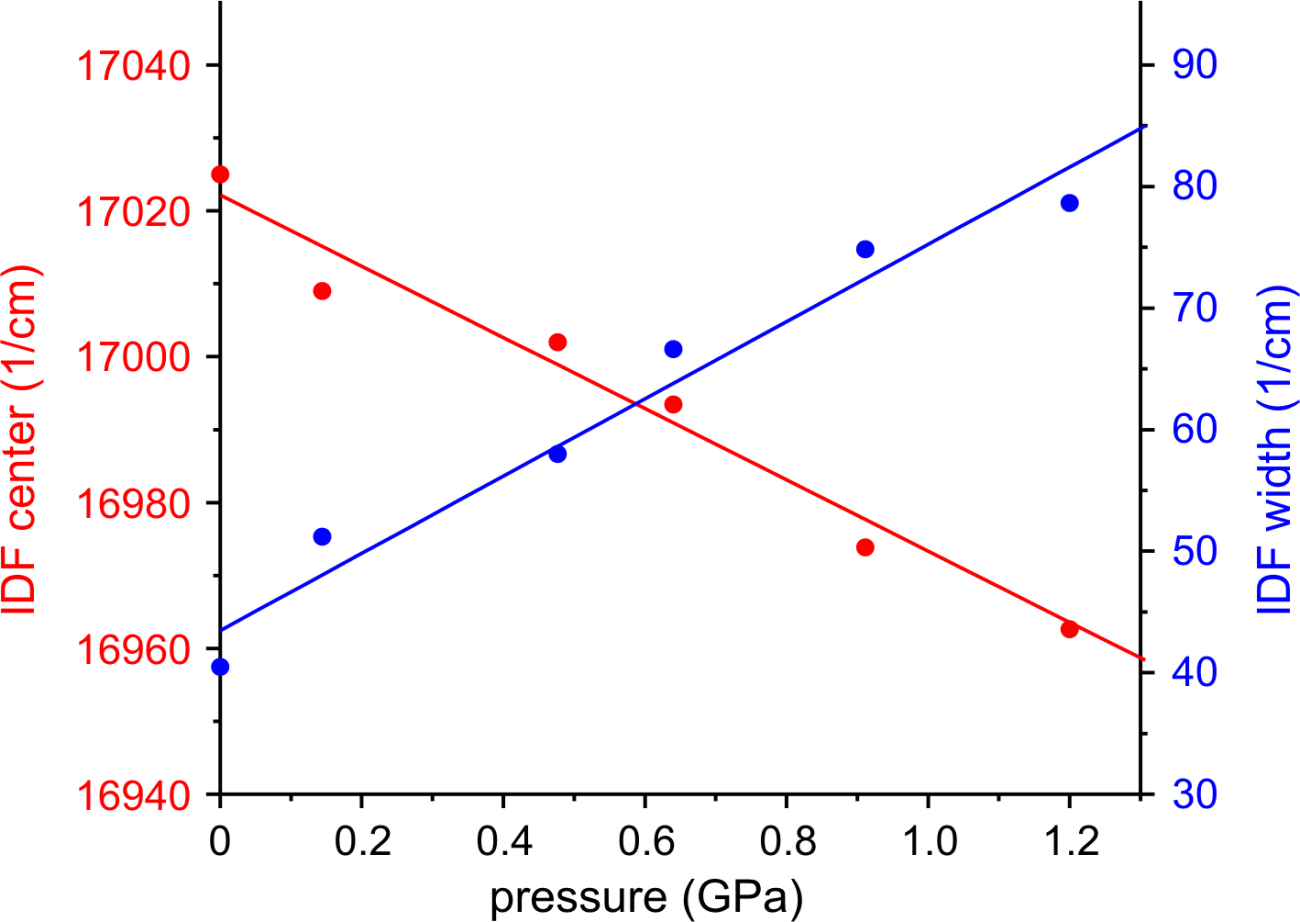
Change of the IDF parameters as a function of pressure in the case of β-Zn-HbA with Cl^-^ as an example. Center and width of the inhomogeneous distribution function (IDF) as a function of applied pressure in the 0–1.2 GPa pressure range are fitted with straight lines. The effect of pressure is of opposite sense on the two parameters.

Upon increasing pressure, red shift and broadening of the IDF was observable both for the hybrid HbAs and for their complexes with heterotropic allosteric effectors. Fig 6 shows these effects in the case of β-Zn-HbA with Cl^-^. We fitted the data points with straight lines according to Eqs 3 and 4. The compressibility was obtained as a parameter of the fitting.

This technique and evaluation procedure was performed on both kinds of samples (α-Zn-HbA and β-Zn-HbA), without and with different allosteric effectors such as chloride ions (Cl^-^), inositol hexaphosphate (IHP), bezafibrate (BZF) or some of their combinations and the respective compressibilities were calculated. These data are summarized in Table 1.

**Table 1:** Compressibilities (*κ*) of HbA under different conditions.

HbA+effectors		*κ* +/- SD (1/GPa)		
α-Zn-HbA	–	**0.17**	+/-	0.05
	Cl^-^	0.19	+/-	0.06
	IHP	0.16	+/-	0.05
	IHP+Cl^-^	0.17	+/-	0.05
	BZF	0.14	+/-	0.04
	BZF+Cl^-^	0.18	+/-	0.06
β-Zn-HbA	–	**0.36**	+/-	0.07
	Cl^-^	0.25	+/-	0.05
	IHP	0.27	+/-	0.05
	IHP+Cl^-^	0.23	+/-	0.05
	BZF	0.19	+/-	0.04
	BZF+Cl^-^	0.15	+/-	0.04

Data of α-Zn-HbA and β-Zn-HbA determined from the pressure shift and broadening of the inhomogeneous distribution functions (IDF-s), without and with different allosteric effectors.

## Discussion

### The compressibility and its change due to the binding of effectors is different in the α and in the β subunit

**Fig 7:**
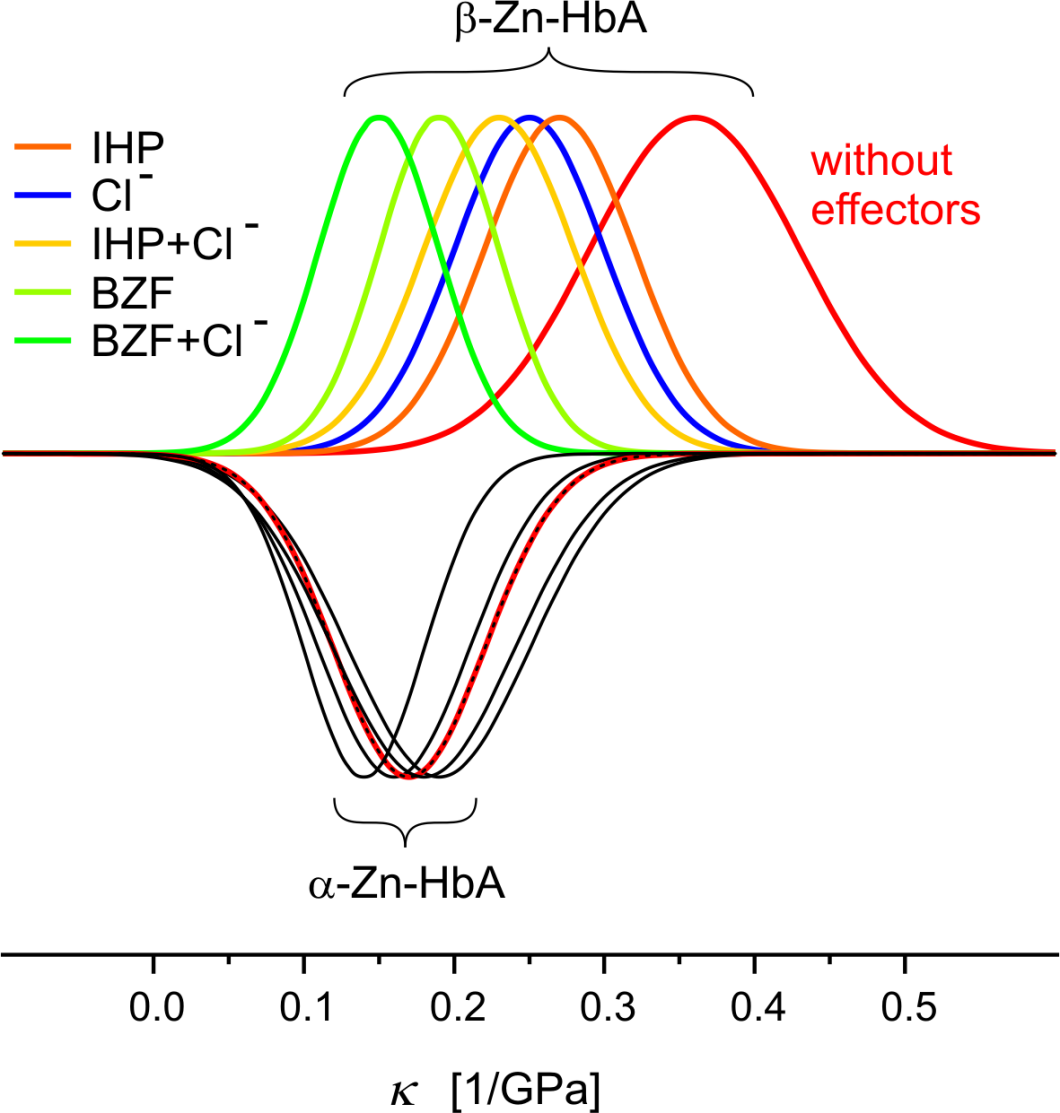
Compressibility data of HbA as “Gaussians”. Curves were created based on the data in Table 1 from the respective *κ* and its standard deviation as parameters of center and width. They were normalized according to their heights. For α-Zn-HbA they are represented as upside down curves to make the data more easily comparable. Red color marks the cases without allosteric effectors, other colors show the influence of different effectors (see in the Figure) and black means no significant difference.

In the interpretation of our results first we compare our data with others measured by different techniques [37, 38]. Based on the theory explained in the Introduction part and on this comparison we consider our compressibilities obtained by FLN as good approximation of the same parameter at room temperature. Our compressibility data are visualized on a more clear-cut form in Fig 7. It is conspicuous that without effectors the compressibility in the β subunit is significantly bigger than in the α subunit (see red curves). It is also visible that in the α subunit the black and red curves generally overlap. This means that there is very slight change in the compressibility due to the binding of heterotropic allosteric effectors. In contrast in the β subunit they cause a significant reduction. IHP (orange) causes the smallest and BZF (light green) the biggest influence. It needs to be mentioned that this trend agrees with that of their efficiency as effectors in lowering the oxygen-binding affinity of hemoglobin [17]. Extra Cl^-^ addition can further intensify the effect in both cases (yellow and green). Unaccompanied Cl^-^ (blue) addition in this concentration can cause similar compressibility reduction as IHP. It is noticeable that in the β subunit the compressibilities maximally decreased by the effectors just reach the values of α subunit. We can read from Table 1 that the compressibility of the β subunit without effectors is 0.36±0.07/GPa, while of the α subunit is about the half, 0.17±0.05/GPa. Their minimal values reached with the effectors are 0.15±0.04/GPa and 0.14±0.04/GPa respectively, which are practically the same as without effectors in the α subunit. We can present the correlation of the compressibility differences and the effector strength (alteration of oxygenation curve based on ref [17]) quantitatively. While the correlation is rather high in the case of the β subunit (r = 0.90), it is very low in the case of the α (r = 0.25).

Before considering and interpreting the meaning of the compressibility data, one needs to consider the conditions of the model that is the basis of deriving these parameters from the experimental data. According to the applied theoretical model for compressibility determination, the system is homogeneous and isotropic with respect to the volume fluctuations [33]. It is clear that a real protein matrix deviates from these conditions. Literature data, however, obtained on proteins show the validity of this approximation in many cases [39, 40]. Another point is that the experimental data refer to the vicinity of the fluorophore, to an “extended” heme pocket (see the transparent colored discs in Fig 2). Therefore, one needs to consider the compressibility data as averages characterizing the conformational dynamics in a surrounding shell of the structure around the hemes. The compressibility data can thus be interpreted as an averaged value based on this volume. According to our estimation, this volume does not exceed the size of one monomer (see Appendix II).

### Relation of compressibility and available structural data

The compressibilities of the α and β subunits were found significantly different. The heme pocket of the α subunit seems much less flexible than that of β. In the amino acid sequence (see Fig 1) we can see that in 63 positions the amino acids are identical (marked with pastel orange rectangles) and 76 are disparate. We also can discover 2–2 local minor deviations because of the absence of amino acids in both cases (marked with green ellipses), but the major, dominant difference is the absence of 5 neighboring amino acids in the α subunit (marked with red ellipse), which practically means the absence of almost the total D segment (see also Fig 2).

As it is visible in Figs 1 and 2 the fluorophore (Zn-PP) can be found between the E and the F helices. Sterically the next closest part to the heme is helix C. This segment is connected to helix E through a loop and segment D. This intermediate section of the amino acid chain is much shorter in the α subunit, thus it may result in a tighter structure in the vicinity of the fluorophore. We believe that such a structural difference may cause a difference in compressibility. That the binding of allosteric effectors decreases the compressibility in the β subunit may derive from a slight conformational effect on the flexible loop between helices C and D altogether with helix D and thus leads to decreased flexibility around the heme. It seems that HbA has a minimal flexibility in its heme pocket structure with a minimal compressibility around 0.15/GPa. The α subunits are almost in this state without bound allosteric effectors, while the β subunits reach it by binding the most efficient effectors.

### Compressibility and the results of molecular dynamics (MD) simulations

MD simulations generally refer to room temperature. In the comparison with our FLN results, we base our argument again on the above discussed similarity of compressibility data obtained at cryogenic and room temperature. Our findings are in accordance with the results of MD simulations of the Perahia group [20, 21, 41]. They found internal cavities in HbA which are not rigid entities; they appear and disappear along the dynamics trajectory. However, some of them persist, although with varying width in both subunits, but there is a persistent cavity in β, which has no equivalent in α. They also studied the possible passageways of a ligand by investigating the displacement of water molecules around the protein. They suggest that describing the water molecules entering or leaving the vicinity of the heme may be a good indicator for the passage of a ligand. What is interesting, it was found that none of water molecules penetrated the α subunits, contrarily to the β where, during the transition, some water molecules started to enter into the heme pocket. The opening of the passage during the transition is due in part to the relative movement of helix D, which has no equivalent in α-chains. The presence of this helix in β gives more flexibility to the region. Their calculations have also shown that the structural changes in the tertiary structure of the β subunit after the quaternary conformational transition plays a prominent role allowing the iron atom to be more accessible to the oxygen.

MD simulations of Hub and co-workers revealed a marked asymmetry between α and β subunits. They described a tendency for β-chains to couple more strongly to the quaternary motion than α-chains [42]. Vesper and de Groot from MD simulations presented a novel allosteric mechanism emphasizing the role of collective dynamics in the global rearrangement of Hb. The first step of the applied approach, (Functional Mode Analysis) is a strict separation of local/intra-chain and global/inter-chain motions. The developed computational technique allowed to find a linear coupling coordinate between tertiary and quaternary motions and thereby to identify allosteric coupling. They also found that the allosteric mechanism of communication between Hb subunits is equally based on hydrogen bonds and steric interactions [43].

According to a new proposed view, binding sites are energetically coupled and a chemical perspective on allostery can be obtained by identifying which amino acid residues are essential for signal propagation and what are the structural or dynamic changes elicited by the excess energy resulting from effector binding. It should be noted that this picture of energy pathways is a simplification, and does not provide a rigorous model of allosteric communication in proteins. Nevertheless, simple pictures of complex phenomena can be extremely powerful [13]. This concept supports the global description of allostery and that the compressibility is a useful parameter in this respect.

### Effector binding sites versus global effect

There are different ideas about the binding sites of heterotropic effectors based on x-ray crystallography and computational methods [3, 18, 44, 45]. The common feature of these sites is that they are usually rather far from the active sites and consequently also from helix C. Hence the effector can cause a tighter structure only because of a global change. The significance of global dynamics is also emphasized in the case of homotropic effectors [46]. The reported structural changes, included displacements of the F helix, which forms the switch region of HbA β subunit and influences the stability of the T state structure. The enhanced global structural fluctuation of the T state can be considered as a”non-site-specific” allosteric effect.

The heterotropic effects of CO_2_ and H^+^ is known as the Bohr effect. Since the released protons also lower the oxygen affinity of Hb, they can be considered as being members of the family of allosteric effectors. Reported results show that chloride ions also contribute to the Bohr effect by neutralizing excess positive charges [47]. Therefore, the Bohr effect, a heterotropic effect, depends on the intricate arrangement and interactions of all hydrogen and anion binding sites in the hemoglobin molecule. It is an excellent example of global electrostatic effects in proteins [48]. Consequently, the shift of the allosteric equilibrium, resulting from the binding of an allosteric effector, arises not only from where the molecule binds but also from how it interacts with Hb [3]. This concept is also supported by the results of pressure-induced tetramer to dimer transition studies resulting in the dissociation constant of the dimer interface [31]. The transition was found to be sensitive to the binding of allosteric effectors showing that conformational changes of the tertiary structure “propagate” from the effector binding sites to the interdimeric interfaces. All these findings support the concept of the “global allostery model” [17].

Based on a novel combination of x-ray diffraction analysis and microspectrophotometric O_2_ equilibrium measurements nine different equilibrium conformers were identified that cover the complete conformational space of hemoglobin, providing clear and convincing evidence of coexisting states [49]. The authors also emphasized that allowing for multiple conformations is not inconsistent with the original MWC model [7], because the number of allosteric states was not restricted by the original model. This was only simplified into a two-state view by many researchers. In the light of these findings the global average compressibility of the subunits (see the transparent colored discs in Fig 2) can be correctly considered as a significant characteristic parameter.

### Functional and dynamic asymmetry of the subunits

Despite of the global structural symmetry of the Hb teramer, it seems that the asymmetry in the α and β subunits of HbA is an indispensable condition of allostery. Authors of the cavity mutant experiments [25] pointed out, that in the O_2_ binding curve of rHb(α) (blue dotted line in Fig 3) the high affinity component is ascribable to the α subunits (detached heme) and the low affinity one to the β subunits (normal heme). They also emphasized that the detached heme in the β subunits (rHb(β), red dotted line in Fig 3) causes an increase of O_2_ affinity also in the α subunits to the same degree as in the other case.

These facts are consonant with the low compressibility or low flexibility of α subunits. We may think that the α subunits have close to optimal but rather rigid structure for O_2_ binding and the presence of Fe-His bonds partially block the binding process reducing the O_2_ affinity in the native HbA. Thus the detachment of Fe-His bonds in the α subunits directly–through tertiary structural changes–increases the O_2_ affinity and the quaternary structure does not change. While when the detachment of Fe-His bonds happens in the β subunits, the same effect can occur just by global, at least by partial quaternary structural changes. It seems that the role of Fe-His binding in general is maintaining cooperativity, while the same bond in the α subunits is responsible for inducing a high-affinity state of the β subunits.

It is also noticeable, that the oxygenation of rHb(α) is slightly influenced by IHP in contrast to the oxygenation of native HbA (blue and green lines as compared to dotted ones in Fig 3), but the low oxygen affinity part of the two curves are very similar. The lowering of oxygen affinity is also observable in the presence of IHP in the upper part of the O_2_ binding curve of rHb(β) (red line in Fig 3). The more rigid but higher affinity α subunits are less sensitive to allosteric effectors, than the β subunits. The *p*_50_ values of this high affinity range are more or less the same in both recombinant Hb-s independently of IHP binding. We found that IHP could not further decrease the compressibility in the α subunits, because it was originally already small.

O_2_ binding of rHb(β) is rather similar to Mb (see Fig 3), which is interesting because rHb(β) is a tetramer, while Mb is a monomer. Nevertheless, the Hill coefficient is *n* = 1.2 which means a low but existing cooperativity. The higher affinity part of native HbA and the rHb(β) is also similar, meaning that the detachment of Fe-His bonds in the β subunits does not cause considerable changes in the same subunits. We think that these findings may also derive from the higher compressibility and thus the possibility for higher volume fluctuations of the β subunits.

### Final conclusions

In this work we applied a unique experimental approach by which the conformational dynamics of the α and the β subunits in HbA could be characterized by the compressibility of the “extended” heme pockets of the size of a subunit. We found that these parameters are different for the two subunits, and are differently modified by the binding of heterotropic allosteric effectors. Our results are in agreement with literature results of molecular dynamics simulations, of structural data and of functional differences of the subunits. We think that compressibility values provide a new characterization parameter of HbA, and that the asymmetry in the response to allosteric effectors suggests a distinct role of the subunits in the response sequence leading to changes in the oxygen binding capability. Based on our results we also suggest a “unified view” composed of the models of Hb allostery based on a variety of approaches as thermodynamics, free energy landscape of population shift, and the structural view of allostery [23]. These descriptions are consistent or at least do not contradict the “global allostery model” [17]. It’s getting clear that in general the solution of the problem of ligand binding is not to find the best fitted special structure for the specific function, but rather, to discover the global character of the process. We believe that this global character must be a synthesis of induced fit and conformational selection [50], that is, strongly based on an emphasis of the role of dynamic conformational ensembles in biomolecular recognition.

### Appendix I

#### Fluorescence line narrowing (FLN) spectroscopy

The basic principles of the FLN technique are the following. Fluorescence emission spectra are measured on samples that are rapidly cooled from room temperature to cryogenic temperatures. As a consequence of the cooling, the thermal fluctuations are practically stopped and the atomic configurations surrounding the chromophore that are populated at room temperature become fixed. This conserved population of dye molecules can be studied by spectroscopy. Every dye molecule has an own electronic transition energy, since it is perturbed by the molecular environment, and every molecular environment of the ensemble has a somewhat “different” structure, which is then reflected in a distribution of the transition energies around a mean value. A subgroup of dye molecules with the “same” or rather “very similar” structural environment means the “same” electronic transition energy of them. With the use of narrow bandwidth (Δ*ν*) excitation, only this specific part of the molecules is selectively excited. Because of the selective excitation and the low temperature, the emission spectra consist of dominant sharp emission lines resulting from resonant excitations superimposed on the background of less dominant broad bands, which are present due to the non-resonant excitation. The m^th^ sharp line originates from the transition between the lowest vibrational level of the first electronic excited state (1, 0) and the ground state (0, 0) after an excitation of (1, m)←(0, 0) and a vibrational relaxation (1, m)→(1, 0) (in general it also called as (0, 0) transition). In a background-subtracted spectrum, the line intensity (*I*_m_) is given by

$$I_{m}=KI_{\mathrm{exc}}AB_{m}N_{m}(\nu ,\Delta \nu )$$ (App1)

where *K* is a constant, *I*_exc_ is the excitation intensity, *A* is the fluorescence emission probability, *B*_m_ is the absorption transition probability, and *N*_m_(*ν*, Δ*ν*) denotes the number of selectively excited molecules at a given excitation frequency (*ν*) and excitation bandwidth (Δ*ν*). In most cases, *N*_m_(*ν*, Δ*ν*) can be given as

$$N_{m}(\nu ,\Delta \nu )=N_{0}\left(n_{m}(\nu +\frac{\Delta \nu }{2})-n_{m}(\nu -\frac{\Delta \nu }{2})\right)$$ (App2)

where *N*_0_ is the total number of the excitable dye molecules and *n*_m_(*ν*) is their cumulative distribution function characteristic for the broadening, which has a same common shape for all the spectral lines (m = 0, 1, 2, …) shifted along the frequency scale. Instead of the cumulative distribution function, we may use the spectral density function which is the derivative of *n*_m_(*ν*)

Nm(ν,Δν)=N0nm'(ν)Δν (App3)

Let us choose the *n*_0_′(*ν*) function which is a possible representation of the so called inhomogeneous distribution function (IDF). Since the source of this broadening is the varying chromophore environment, this function is characteristic for the distribution of chromophore molecules in their environments [51].

### Appendix II

#### Estimation of the volume in which the compressibility is measured

Starting with Eq 2 we consider how far the interaction of the chromophore and the surrounding atomic groups–leading to the solvent shift *ν*_s_−can extend. The interaction potential is often assumed being inversely proportional to the intermolecular distance (*r*) between the chromophore and the interacting solvent molecule by a power function [52]. Thus

$$\nu_{s}(r)=\alpha r^{-n},$$ (App4)

where *α* is a proportionality constant. A reasonable choice for *n* is 6, as is the case for dispersion or higher order electrostatic interactions (attractive long-range part of the Lennard-Jones potential). We have to remark that in our experiments instead of a classical solvent shift, we have a “matrix-shift” caused by the interaction with the protein, but for simplicity hereafter we also use the term “solvent”. Let us consider a spherical volume around the chromophore with radius *r*. This environment (matrix) is assumed to be homogeneous and isotropic; and each solvent molecule interacts independently with the chromophore [53, 54]. Since we expect that an ensemble of solvent molecules may influence the transition frequency of the chromophore, we write the mean solvent shift an integral form:

$$\langle \nu_{s}\rangle =\int_{V_{0}}^{\infty }\alpha r^{-6}g(r)dr\cdot d\vartheta \cdot d\phi =\int_{R_{0}}^{\infty }\alpha r^{-6}g(r)4\pi r^{2}dr$$ (App5)

where angle brackets indicate the expected value, the lower limit of the integral (*V*_0_) is the (effective) spherical volume of the chromophore, *g*(*r*) is standing for the kernel function, which may include arbitrary weighing along the distance, for the sake of simplicity we assume here a uniform distribution of the solvent molecules and *dr·dϑdφ* = *dV* is the volume element in the polar coordinate system. In the last part of the equation, *R*_0_ is the (effective) radius of the chromophore and 4*πr*^2^ is the result of the integral according to *dϑ* and *dφ*.

Denote *R*_max_ the radius of the spherical volume in which the compressibility is measured. Theoretically *R*_max_ = ∞, because the interaction of Lennard-Jones potential is infinitely long range. Fortunately, this interaction falls off with distance rather quickly. Let us calculate what error (*P*) is caused if we limit *R*_max_ to a finite value. To do this, we define *P* as the ratio of the contribution of the solvent shift outside of this spherical volume (*ν*_s(out)_) for *r* > *R*_max,_ and that for the total range. We may also use this definition in the opposite direction: If we choose an arbitrary *P* value we can determine the respective *R*_max_ as *R*_(1-*P*)_, the radius of the sphere which can be considered for *R*_max_ as an approximation with *P* error.

$$P=\frac{\nu_{s(\mathrm{out})}}{\langle \nu_{s}\rangle }=\frac{\int_{R_{\left(1-P\right)}}^{\infty }r^{-4}dr}{\int_{R_{0}}^{\infty }r^{-4}dr}=\frac{{\left[r^{-3}\right]}_{R_{\left(1-P\right)}}^{\infty }}{{\left[r^{-3}\right]}_{R_{0}}^{\infty }}={\left(\frac{R_{\left(1-P\right)}}{R_{0}}\right)}^{-3}.$$ (App6)

Let be *P* in succession 1%, 2% and calculate *R*_99%_, *R*_98%_ according to

$$R_{\left(1-P\right)}=R_{0}P^{-\frac{1}{3}}.$$ (App7)

From structural data we estimate the volume of the central part of a heme group to be approximately 300Å^3^ and thus in the simple spherical approximation we can take *R*_0_ as [(0,75/π)·300]^1/3^ ≈ 4.2 Å. Thus *R*_98%_ ≈ 15 Å, *R*_99%_ ≈ 19 Å (see Fig 2). The *P* value can be estimated from the accuracy of the parameter estimation from experimental data. In our experiments the uncertainity of our measuring procedure is about 1–2%, therefore we take *P* in this range. This suggests that in the case of FLN experiments we can assume that the compressibility values are referring to approximately the size of a single monomer. We found several demonstrative evidence consistent with this approach (see Discussion section).

### Appendix III

#### A concise overview on Hb’s allosteric models

The classical description of allostery in O_2_ binding has been derived from the concept that Hb as a tetramer exists in equilibrium between two conformations of distinct oxygen affinity, the T (tense) and the R (relaxed) states. In the two-state allosteric model, Monod, Wyman, and Changeau (MWC) assigned the fully deoxygenated Hb to the T and fully oxygenated Hb to the R states. Cooperativity of oxygen binding arises from a shift in the population from the low-affinity T state to the high-affinity R state as the oxygen concentration is increased. Thus the binding of O_2_ alters the relative stabilities of the T and R states, whereas the O_2_ affinity of the four subunits remains constant at each stage of ligation [7–9]. The two-state model was given an explicit structural interpretation by Perutz. He proposed a stereochemical model of Hb to structurally describe the mechanistic details of the cooperativity on the basis of the molecular structures of deoxy- and oxy-Hbs. He assumed a structure-function correlation and assigned the T and R quaternary structures to low-affinity and high-affinity functional states [10]. For decades, the other dominant model for allostery was the ‘sequential’, or KNF (Koshland–Nemethy–Filmer), model [11]. This model assumes an induced-fit mechanism where ligand binding to one domain triggers conformational changes in the other domain, and can thus be easily understood in terms of the propagation of an interdomain signal. The rather new ensemble allostery model (EAM) provides a generalization of both models, allowing for different conformational states to be populated [12]. A recent review article highlights advances in describing structural and dynamic changes in allosteric proteins, the identification of residues important for signal propagation, the significance of the concept of allosteric pathways and how a chemically appealing picture of allostery can be obtained [13].

The MWC model has correctly interpreted several properties of cooperativity in hemoglobin O_2_ binding and it ascribes the heterotropic effects to shifts of the allosteric equilibrium between the two quaternary structures, however Imai [14] showed that these heterotropic effects cannot be realized without assuming that the oxygen affinity of either quaternary structure had been influenced by the bound heterotropic effectors. The tertiary two state model (TTS model) [15] was proposed to solve this inconsistency while conserving the essence of the MWC model.

Extended studies on the effect of heterotropic allosteric effectors indicated that they do not only bind to the T-state but also to the R-state. The modulation of the oxygen association constants in both states was shown to occur at a much broader scale than previously known. These data initiated the reformulation of the earlier models for the allosteric action of heterotropic effectors [16, 17]. The proposed “global allostery model” supposes that these effectors can bind to both the T- and R-states inducing direct tertiary conformational changes. Former models supposed a change of the interfaces between the subunits, which is a quaternary effect, but not a change in the tertiary structures of the subunits themselves. In this context, it is an important question how the proposed changes in the tertiary structure propagate from the interdimeric interfaces to the oxygen-binding site. It seems that conformational dynamics of the subunits may be the property that couples the interfacial tertiary changes to the site of the regulation of oxygen-binding. Moreover, the tertiary changes may very well be not only static structural rearrangements, but also changes in the dynamics and mobility of the oxygen binding site and its surroundings [20, 21].

Several new models describing the action of allosteric effectors on hemoglobin function have been published in the literature. The question of allostery without a conformational change has been raised by Tsai and Nussinov. They suggested the thought that ‘not observed does not imply that it is not there’ and they emphasize that there can be several possible reasons for the apparent missing evidence for conformational change [22]. They also presented a unified view about allostery [23]. Many models have been successfully formulated, and are able to describe allostery even in the absence of a detailed structural mechanism.
